# Effect of a multidisciplinary approach on hospital visit continuation in the treatment of patients with alcohol dependence

**DOI:** 10.1002/npr2.12349

**Published:** 2023-05-22

**Authors:** Tsutomu Kurata, Tasuku Hashimoto, Hitoshi Suzuki, Minoru Ishige, Shuichi Kikuchi

**Affiliations:** ^1^ Department of Psychiatry Sodegaura Satsukidai Hospital Sodegaura Japan; ^2^ Department of Psychiatry, School of Medicine International University of Health and Welfare Chiba Japan; ^3^ Department of Psychiatry, Graduate School of Medicine Chiba University Chiba Japan

**Keywords:** alcohol abstinence, alcohol dependence, alcoholism treatment program, continuous treatment, multidisciplinary approach

## Abstract

**Aims:**

Given the high dropout rates from initial treatment for alcoholism among patients with alcohol dependence, it is highly essential to prevent alcohol‐dependent patients from early dropout. This study aims to investigate whether a multidisciplinary approach can help achieve continuous hospital visits for this patient population for initial treatment.

**Methods:**

This is a retrospective cohort study based on the medical records of all sequential alcohol‐dependent outpatients who visited Sodegaura Satsukidai Hospital for alcoholism at least once between October 2017 and March 2019. The primary outcome was the difference in the rates of patients who achieved 6 and 12 months of continuous hospital visits following the first visit with and without the multidisciplinary approach.

**Results:**

Of all the participants (*n* = 67), the female‐to‐male ratios for patients supported with and without the multidisciplinary approach were 6:30 and 5:26, respectively. It was found that the rate of alcoholic patients treated with the multidisciplinary approach (*n* = 33, 91.7%), who had continuous hospital visits, was significantly higher than that of those without (*n* = 12, 38.7%) (*χ* ^2^ = 21.2, *p* < 0.0001) during the first 6 months of treatment. Similarly, the rate of alcoholic patients treated with the multidisciplinary approach (*n* = 29, 90.6%) having continuous visits was significantly higher than that of those who did not receive such support (*n* = 8, 25.8%) (*χ* ^2^ = 27.3, *p* < 0.0001) during the first 12 months.

**Conclusion:**

A multidisciplinary approach can be used to reduce dropout from initial treatment among outpatients with alcohol dependence.

## Abstract 2

This study was aimed to investigate whether a multidisciplinary approach can help achieve continuous hospital visits for this patient population for initial treatment through the retrospective design using medical records of all sequential alcohol‐dependent outpatients in a hospital (Japan) between October 2017 and March 2019. The rate of alcoholic patients treated with the multidisciplinary approach who had continuous hospital visits, was significantly higher than that of those without during both the first 6 and 12 months of treatment.

## Graphical abstract

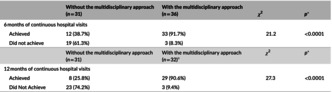

## 1 INTRODUCTION

Almost 10% of the world's population is afflicted with alcohol use disorders; yet, the treatment of alcohol dependence remains a challenge.^1^ Moreover, many patients with alcohol dependence are likely to drop out of their initial treatment for alcoholism or do not complete the treatment program.^2^ Adherence to treatment for alcoholism is associated with physical and psychological outcomes.^2^ Heavy users of alcohol are more likely to drop out of addiction treatment programs.^3^ To properly address the alcohol‐dependence problem, it is important to ensure that patients keep visiting the clinic for treatment without dropping out. In the treatment of substance use disorders, those who receive a longer treatment show better abstinence results; thus, treatment duration is significantly associated with substance use status.^4^ If the patients wish to stay away from the hospital and drop out of treatment, we as medical professionals have no right to intervene. As a result, patients who struggle to seek help may lose their health, their family, their job, or even die. Therefore, it is important to develop strategies to prevent alcoholic patients from dropping out of treatment.

Regarding practical treatment strategies for alcoholism, Dionisi and colleagues reported that these patients may benefit greatly from a multidisciplinary treatment program.^5^ Moreover, evidence shows that a multidisciplinary approach is effective in the provision of optimal treatment of alcoholic liver disease caused by alcohol use disorders.^6^, ^7^ However, few studies have reported the effectiveness of multidisciplinary approaches on continuous hospital visits in the treatment of alcohol dependence.

Accordingly, this study aims to investigate whether a multidisciplinary approach can contribute to continuous hospital visits of outpatients with alcohol dependence for treatment. We hypothesized that a multidisciplinary approach would be an important factor in improving continued hospital visits and, thus, recovery from alcohol dependence. Therefore, we conduct a retrospective study of our sample consisting of alcohol‐dependent patients.

## 2 METHODS

### 2.1 Study design and participants

This is a retrospective cohort study that is based on the medical records of all sequential out‐patients with alcohol dependence who met the following inclusion criteria: (1) they visited the outpatient clinic for alcoholism at Sodegaura Satsukidai Hospital at least once between October 2017 and March 2019; (2) they were diagnosed with alcohol dependence by expert psychiatrists according to the ICD‐10.

### 2.2 Data collection

The medical records of the participants included information on the following aspects: age, gender, a multidisciplinary approach for the treatment of alcohol dependence, an inpatient treatment program for alcohol dependence, period of alcohol dependence, comorbid mental illnesses, presence or absence of a spouse, durations of abstinence after the start of the treatment and those of continuous hospital visits, medical drugs for alcohol dependence, the scores of the alcohol use disorders identification test (AUDIT), values of γ‐glutamyl transpeptidase (GGTP), aspartate aminotransferase (AST), and alanine aminotransferase (ALT) recorded in the first visit.

### 2.3 Definition of continuous hospital visits and alcohol abstinence or consumption

In this study, participants achieving continuous hospital visits during the first 6 and 12 months of alcoholic treatment are those who regularly and continuously visited the outpatient clinic of the hospital for alcohol dependence treatment after their first visit for those particular periods. Alcohol abstinence during those periods was measured based on the subjective or objective reports of patients regarding their abstinence performance,^8^ as well as laboratory liver function tests (GGTP, AST, and ALT). Alcohol consumption after abstinence was determined based on reports of outpatients, their families, or attendants, visiting nurses, and obviously laboratory liver function tests.

To accurately assess patients' continuous hospital visits, we excluded patients who died or were transferred to other hospitals during the study period. Similarly, such patients were also excluded for examining alcohol abstinence or consumption after abstinence.

### 2.4 The intervention with a multidisciplinary approach for the treatment of patients with alcohol dependence

Our research institute, Sodegaura Satsukidai Hospital, provides alcoholism treatment programs for in‐ and outpatients using a multidisciplinary approach. For both these programs, the group cognitive behavioral therapy (CBT) based on Serigaya methamphetamine relapse prevention program (SMARPP),^9^ that is available not only for drug addiction treatment but also for the alcoholism treatment program, has been implemented as the main psychological treatment. The inpatient alcohol rehabilitation program is provided by a multidisciplinary team consisting of nurses, occupational and physical therapists, social workers, nutritionists, pharmacists, and physicians. The program includes detailed medical checkups and treatment, group CBT, nutritional and psychosocial instruction, and support for inpatients and their families during the 1–2 months of hospitalization at the meeting room or occupational therapy space of the hospital. The group therapy sessions take place once a week, with about 5–10 participants per session. The contents of group therapy for inpatients include brief CBT and alcohol abstinence education such as “Advantages and Disadvantages of Drinking,” “Triggers and Cravings,” and “Refusing Invitations to Drink.”

Likewise, the outpatient treatment program consists of group CBT, occupational therapy, and social support to initiate and maintain alcohol abstinence and prevent the interruption of outpatients' hospital visits. The outpatient program team also consists of social workers, nurses, psychiatrists, and occupational therapists. Outpatient group therapy is offered twice a month, with approximately 15 participants each time. Outpatient group therapy addresses not only CBT and abstinence education, but also offers recreation activities such as physical exercise, craft work, and cooking. The outpatient treatment program with a multidisciplinary approach is implemented from 10:00 to 11:30 a.m. every other Friday and from 1.30 to 2.30 p.m. every other Tuesday at the hospital.

Regarding the role of each expert member in a multidisciplinary approach, nurses not only provide care for patients with alcoholic and alcohol‐related diseases, but also help the patients live a healthy life including abstinence and regularly taking medication; occupational therapists help alcoholic patients improve their social abilities and learn to use time effectively to abstain from alcohol consumption through occupational programs; physical therapies not only treat inpatients with physical disabilities such as Wernicke's encephalopathy and peripheral neuropathy, but also provide instruction on maintaining physical function, especially in the inpatient program; social workers set up and support social problems that patients and their families face in everyday life and facilitate the in/outpatient alcoholism treatment program; nutritionists are involved in the inpatient program as a member of the nutrition support team and also provide regular lectures and consultations on alcohol‐related nutritional deficiencies and healthy and sustainable diet; pharmacists instruct patients on medication administration and pharmacotherapy for alcoholism; physicians manage the alcoholism treatment programs for in‐ and outpatients using a multidisciplinary approach.

Basically, we initially treat alcoholic patients in outpatient settings. We recommend the cases who fail to abstain from alcohol or cannot stop drinking in the settings, to participate in the inpatient alcohol rehabilitation program with a detailed explanation. Some patients voluntarily participate in the inpatient program following their involuntary admission due to exacerbation of their psychiatric conditions such as alcohol withdrawal delirium.

### 2.5 Definition of the two groups: Alcoholic patients treated with and without a multidisciplinary approach

All the consecutive patients were recommended to participate in the in‐ and/or outpatient alcoholism treatment programs at least once by their physicians. In this study, the alcoholic patients who participated in the in‐ or outpatient treatment programs of the institute at least once were assigned to the intervention group with a multidisciplinary approach. Conversely, the patients who never participated in the alcoholism treatment program in the in‐ and outpatient treatment programs of the hospital were assigned to the intervention group without a multidisciplinary approach. The patients without multidisciplinary interventions received psychotherapy, including psychoeducation, motivational interviewing, and brief cognitive behavioral therapy and pharmacotherapy, only by a psychiatrist without a multidisciplinary approach. Therefore, other medical staff were not involved with these patients at all.

### 2.6 Ethical consideration

This study was approved by the Ethics Committee of Sodegaura Satsukidai Hospital in Sodegaura City, Japan. The committee waived the requirement for approval and written informed consent for patient participation, as the present study was retrospective in nature where only anonymized data were used to protect the privacy of the participants by maintaining confidentiality. Information regarding this study was posted in the outpatient department and daycare of Sodegaura Satsukidai Hospital to notify participants of the opt‐out option. The present study was conducted in accordance with the Helsinki Declaration.

### 2.7 Primary and secondary outcomes

The primary outcome of this study was the difference in the rates of alcoholic patients who achieved 6 and 12 months of continuous hospital visits after their first visit between two groups, one with a multidisciplinary approach and the other without. The secondary outcomes were to identify any difference in the rates of patients achieving 6 and 12 months of alcohol abstinence between these two groups, and to explore differences in continuous hospital visits and alcohol abstinence between patients using only the outpatient multidisciplinary program and those without any multidisciplinary program. As the other secondary outcomes, we also explored effects of clinical characteristics such as psychiatric comorbidities, medication, and other demographic data for alcoholism on continuous hospital visits and abstinence in patients with alcohol dependence.

### 2.8 Statistical analyses

We analyzed the data pertaining to the alcohol‐dependent patients supported with and without a multidisciplinary approach separately. All the analyses were conducted using the statistical package for the social sciences (SPSS) version 23.0 (IBM, NY, US). The analyses of the primary and secondary outcomes were performed using the chi‐square or Fisher's exact tests. We also employed the chi‐square or Fisher's exact tests for categorical variables and Student's *t*‐tests for continuous variables in the demographic data. The level of significance was set at *p* < 0.05.

## 3 RESULTS

### 3.1 Participant characteristics

Table 1 shows the characteristics of the participants in this study (*n* = 67). On the one hand, of the 36 alcoholic patients supported with a multidisciplinary approach, 26 participated in both the in‐ and outpatient programs, while 8 participated only in the outpatient program and 2 only in the inpatient program. The shortest admission period during which the patients were assigned to the inpatient treatment program was 25 days in this study. On the other hand, 31 patients did not participate in either the in‐ or outpatient programs. The main reason the patients did not even participate in the outpatient treatment program was that they were not good at attending in‐group meetings. The other reasons for not joining the outpatient program were that patients' work schedule did not fit with the program, or those without complete sobriety felt uncomfortable to join the program. Only one alcoholic patient who was treated with the multidisciplinary approach used the drug addiction recovery center (DARC) and no one used narcotics anonymous (NA) in this study. Six patients participated in alcoholics anonymous (AA) in this study. Among them, five patients were treated with the multidisciplinary approach, and one was treated without it.

**TABLE 1:** Characteristics of alcoholic patients treated with and without a multidisciplinary approach.

Characteristics	Without the multidisciplinary approach (*n* = 31)	With the multidisciplinary approach (*n* = 36)
Age, mean (SD)	58.8 (15.2)	55.8 (12.5)
Sex, *n* (%)		
Male	26 (83.9)	30 (83.3)
Female	5 (16.1)	6 (16.7)
Using the in‐ and outpatient multidisciplinary program, *n* (%)	0 (0)	26 (72.2)
Using only the inpatient multidisciplinary program, *n* (%)	0 (0)	2 (5.6)
Using only the outpatient multidisciplinary program, *n* (%)	0 (0)	8 (22.2)
Using neither the in‐ or outpatient multidisciplinary program, *n* (%)	31 (100)	0 (0)
Length of dependence, *n* (%)		
Less than 10 y	25 (80.6)	26 (72.2)
10 y or more	6 (19.4)	10 (27.8)
With spouse, *n* (%)	16 (51.6)	15 (41.7)
Treated without Acamprosate, *n* (%)	8 (25.8)	4 (11.1)
Treated with Acamprosate, *n* (%)	23 (74.2)	32 (88.9)
Without Disulfiram or Cyanamid, *n* (%)	21 (67.7)	11 (30.6)
With Disulfiram or Cyanamid, *n* (%)	10 (32.3)	25 (69.4)
AUDIT, mean (SD)	24.7 (7.6)	29.1 (6.6)
GGTP, mean (SD), U/L	169.6 (319.7)	327.8 (372.0)
AST, mean (SD), U/L	47.5 (45.7)	86.2 (61.0)
ALT, mean (SD), U/L	30.0 (34.8)	44.8 (43.6)
Other alcohol‐related diagnoses		
Alcohol withdrawal syndrome	0	8
Alcoholic psychosis	0	2
Alcohol amnestic disorder	2	2
Korsakoff syndrome	0	1
Comorbid neuropsychiatric diseases		
Dementia in Alzheimer's disease	1	0
Amphetamine dependence	1	0
Delusional disorder	1	0
Bipolar affective disorders	3	10
Depressive disorders	2	1
Anxiety disorders	1	2
Personality disorder	1	0
Eating disorders	1	1
Symptomatic epilepsy	0	1
Attention‐deficit/hyperactivity disorder	1	0

Abbreviations: ALT, alanine aminotransferase; AST, aspartate aminotransferase; AUDIT, Alcohol Use Disorders Identification Test; GGTP, γ‐glutamyl transpeptidase.

The AUDIT and AST values were significantly lower in the group without the multidisciplinary approach than that with it (AUDIT, *t* (64.0) = 2.5, *p* = 0.015; AST, *t* (63.3) = 2.9, *p* = 0.005), although there were no differences in GGTP and ALT values among the two groups.

### 3.2 Effect of the multidisciplinary approach on continuous hospital visits

Of the 67 participants, 45 achieved continuous hospital visits during the first 6 months of the alcoholism treatment, while the remaining 22 did not. Among these 45 patients, the percentage of alcoholic patients treated with the multidisciplinary approach (*n* = 33, 91.7%) was significantly higher than that of those without (*n* = 12, 38.7%) (*χ* ^2^ = 21.2, *p* < 0.0001; Table 2). Similarly, among the patients who achieved continuous hospital visits during the first 12 months of treatment (*n* = 37), the percentage of patients treated with the multidisciplinary approach (*n* = 29, 90.6%) was significantly higher than that of those without (*n* = 8, 25.8%) (*χ* ^2^ = 27.3, *p* < 0.0001; Table 2). Four patients were excluded from the first 6 and 12 months of treatment, as three died and one was transferred to another hospital.

**TABLE 2:** Continuous hospital visits among the alcoholic patients treated with and without the multidisciplinary approach.

	Without the multidisciplinary approach (*n* = 31)	With the multidisciplinary approach (*n* = 36)	*χ* ^2^	*p* *
6 months of continuous hospital visits				
Achieved	12 (38.7%)	33 (91.7%)	21.2	<0.0001
Did not achieve	19 (61.3%)	3 (8.3%)		

	Without the multidisciplinary approach (*n* = 31)	With the multidisciplinary approach (*n* = 32)^a^	*χ* ^2^	*p* *
12 months of continuous hospital visits				
Achieved	8 (25.8%)	29 (90.6%)	27.3	<0.0001
Did Not Achieve	23 (74.2%)	3 (9.4%)		

^*^ *p*‐values were calculated by the chi‐squared test.

^a^ Four patients were lost during the 6–12 months period after the first 6 months of treatment for alcohol dependence since three patients died and one was transferred to another hospital.

In addition, regarding alcoholic patients using only the outpatient multidisciplinary program (only outpatients' program users), the rates of achievers of continuous hospital visits in the only outpatients' program users were significantly higher than the rates in those without any multidisciplinary program during the first 6 months (used by the Fisher's exact test, *p* = 0.003) and first 12 months of treatment (the Fisher's exact test, *p* = 0.016; Table 3).

**TABLE 3:** Continuous hospital visits among the alcoholic patients using only the outpatient multidisciplinary program and those using neither the in‐ or outpatient multidisciplinary program.

	Using neither the in‐ or outpatient multidisciplinary program (*n* = 31)	Using only the outpatient multidisciplinary program (*n* = 8)	*p* *
6 months of continuous hospital visits			
Achieved	12 (38.7%)	8 (100%)	0.003
Did not achieve	19 (61.3%)	0 (0%)	

	Using neither the in‐ or outpatient multidisciplinary program (*n* = 31)	Using only the outpatient multidisciplinary program (*n* = 8)	*p* *
12 months of continuous hospital visits			
Achieved	8 (25.8%)	6 (75.0%)	0.016
Did Not Achieve	23 (74.2%)	2 (25.0%)	

* Fisher's exact test was conducted.

### 3.3 Effect of the multidisciplinary approach on alcohol abstinence

It was found that 59 outpatients with alcohol dependence achieved alcohol abstinence during the first 6 months of treatment. Eight patients were excluded since they did not visit the hospital after the first time. Among these 59 patients, the rate of alcoholic patients treated with the multidisciplinary approach (*n* = 14, 38.9%) was significantly higher than that of those without (*n* = 3, 13.0%) (*χ* ^*2*^ = 4.6, *p* < 0.033; Table 4).

**TABLE 4:** Alcohol abstinence among the alcoholic patients treated with and without the multidisciplinary approach.

	Without the multidisciplinary approach (*n* = 23)	With the multidisciplinary approach (*n* = 36)	*χ* ^2^	*p* *
6 months of alcohol abstinence				
Achieved	3 (13.0%)	14 (38.9%)	4.6	0.033
Did not achieve	20 (87.0%)	22 (61.1%)		
Cases lost after the first hospital visit	8	4		

	Without the multidisciplinary approach (*n* = 21)	With the multidisciplinary approach (*n* = 31)	*χ* ^2^	*p* *
12 months of alcohol abstinence				
Achieved	2 (9.5%)	11 (35.5%)	4.5	0.034
Did not achieve	19 (90.5%)	20 (64.5%)		
Cases lost between 6 and 12 months after the first visit	10	2		
Confirmed patient deaths between 6 and 12 months after the first treatment	0	3		

* *p*‐values were calculated by the chi‐squared test.

A total of 52 patients with alcohol dependence were identified to assess alcohol abstinence during the 12 months of alcoholic treatment. Four patients were lost between 6 and 12 months of treatment. Furthermore, three died of diseases during this period. The rate of the patients treated with the multidisciplinary approach (*n* = 11, 35.5%) was found to be significantly higher than the rate of those without (*n* = 2, 9.5%) (*χ* ^*2*^ = 4.5, *p* < 0.034; Table 4).

In addition, regarding only outpatients' program users, there were no differences between the rates of achievers of alcohol abstinence in the only outpatients' program users and those in the alcoholic individuals without any multidisciplinary program during the first 6 months and 12 months of treatment (Table 5).

**TABLE 5:** Alcohol abstinence among the alcoholic patients using only the outpatient multidisciplinary program and those using neither the in‐ or outpatient multidisciplinary program.

	Using neither the in‐ or outpatient multidisciplinary program (*n* = 23)	Using only the outpatient multidisciplinary program (*n* = 8)	*p* *
6 months of alcohol abstinence			
Achieved	3 (13.0%)	4 (50.0%)	0.053
Did not achieve	20 (87.0%)	4 (50.0%)	
Cases lost after the first hospital visit	8	0	

	Using neither the in‐ or outpatient multidisciplinary program (*n* = 21)	Using only the outpatient multidisciplinary program (*n* = 7)	*p* *
12 months of alcohol abstinence			
Achieved	2 (9.5%)	3 (42.9%)	0.082
Did not achieve	19 (90.5%)	4 (57.1%)	
Cases lost or transferred to other hospital between 6 and 12 months after the first visit	10	1	

* Fisher's exact test was conducted.

### 3.4 Effects of medication for alcoholism on and abstinence

We additionally analyzed the differences in abstinence rates between alcoholic patients treated with and without disulfiram, cyanamide, or acamprosate in this study. Consequently, there were no differences among them.

### 3.5 The relationship between clinical characteristics and alcohol abstinence among each alcoholic group with or without the multidisciplinary approach

By assessing any difference in demographic data, including age, sex, anti‐alcoholic medication, spouses, initial AUDIT scores, and the data of initial laboratory liver tests (GGTP, AST, ALT), between the patients who achieved alcohol abstinence and those who did not among those treated with a multidisciplinary approach, no significant difference was found among the two groups. Additionally, regarding patients without the multidisciplinary approach, there were no differences in clinical characteristics between the patients who achieved alcohol abstinence and those who did not.

### 3.6 Effect of psychiatric comorbidities in the alcoholic patients on continuous hospital visits and alcohol abstinence

Regarding the number of comorbid neuropsychiatric patients, three patients suffered from two comorbidities, and one suffered from three comorbid diseases in this study. Among the patients without comorbidities but not those with comorbid neuropsychiatric diseases, there were significant differences in the rate of continuous hospital visits of 6 and 12 months: the percentages of alcoholic patients treated with the multidisciplinary approach were higher than those of patients treated without it (Table 6). Regarding alcohol abstinence, the results were similar, that is, the percentages of non‐comorbid patients with the multidisciplinary approach were significantly higher than those without it, although there were no differences among patients with comorbid neuropsychiatric diseases (Table 7).

**TABLE 6:** Effect of psychiatric comorbidities in the alcoholic patients on continuous hospital visits.

Non‐comorbidities			*χ* ^2^ [d.f.]	*p* ^a^
	Without the multidisciplinary approach (*n* = 22)	With the multidisciplinary approach (*n* = 24)		
6 months of continuous hospital visits				
Achieved	4 (18.2%)	23 (95.8%)	28.5 [1]	<0.001*
Did not achieve	18 (81.8%)	1 (4.2%)		

	Without the multidisciplinary approach (*n* = 22)	With the multidisciplinary approach (*n* = 21)		
12 months of continuous hospital visits				
Achieved	3 (13.6%)	19 (90.5%)	25.4 [1]	<0.001*
Did Not Achieve	19 (86.4%)	2 (9.5%)		

Comorbid neuropsychiatric diseases			*p* ^b^
	Without the multidisciplinary approach (*n* = 9)	With the multidisciplinary approach (*n* = 12)	
6 months of continuous hospital visits			
Achieved	8	10	1.0
Did Not Achieve	1	2	

	Without the multidisciplinary approach (*n* = 9)	With the multidisciplinary approach (*n* = 11)	
12 months of continuous hospital visits			
Achieved	5	10	0.127
Did Not Achieve	4	1	

Abbreviation: *d.f*.: degrees of freedom.

^a^ *p*‐values were calculated for the results of the *χ* ^2^‐test.

^b^ *p*‐values were calculated for the results of Fisher's exact test.

* *p* < 0.001.

**TABLE 7:** Effect of psychiatric comorbidities in the alcoholic patients on alcohol abstinence.

Non‐comorbidities			*p* ^a^
	Without the multidisciplinary approach (*n* = 15)	With the multidisciplinary approach (*n* = 24)	
6 months of alcohol abstinence			
Achieved	0 (0.0%)	10 (41.7%)	0.006**
Did not achieve	15 (100%)	14 (58.3%)	

	Without the multidisciplinary approach (*n* = 14)	With the multidisciplinary approach (*n* = 20)	
12 months of alcohol abstinence			
Achieved	0 (0.0%)	7 (35.0%)	0.026*
Did Not Achieve	14 (100%)	13 (65.0%)	

Comorbid neuropsychiatric diseases			*p* ^a^
	Without the multidisciplinary approach (*n* = 7)	With the multidisciplinary approach (*n* = 11)	
6 months of alcohol abstinence			
Achieved	2	4	1.0
Did Not Achieve	5	7	

	Without the multidisciplinary approach (*n* = 9)	With the multidisciplinary approach (*n* = 11)	
12 months of alcohol abstinence			
Achieved	5	10	0.127
Did Not Achieve	4	1	

^a^ *p*‐values were calculated for the results of Fisher's exact test.

* *p* < 0.05

** *p* < 0.01.

## 4 DISCUSSION

Two important findings were obtained in this study. First, the percentage of alcoholic patients treated with a multidisciplinary approach who continued to visit the hospital was significantly more than that of those treated without a multidisciplinary approach, regardless of whether they were engaged in an inpatient or outpatient program. Second, the study showed that alcoholic patients who received the multidisciplinary intervention were more likely to continue to abstain from alcohol than those who did not.

Alcoholic patients who received multidisciplinary intervention from a treatment program could continue to visit the hospital more than those who did not. Regarding the effectiveness of a multidisciplinary approach for the treatment of alcoholism, Avila et al. (2008) reported that the rate of continuous hospital visits is higher when both psychiatrists and internal medicine specialists are involved in the treatment process as compared to only psychiatrists.^10^ Their study revealed that a multidisciplinary approach among specialized medical doctors is effective for alcoholic treatment. Although the previous report's findings^10^ are consistent with ours, the present study highlights the importance of the involvement of not only medical doctors but also professionals from different fields (e.g., social workers, nurses, and occupational therapists) in preventing treatment dropouts in patients in order to achieve recovery from alcohol addiction. Additionally, it was found that people with an alcohol‐related liver disease who were treated by a multidisciplinary team of professionals with unified expertise achieved a higher number of discharges, which is consistent with the findings of a study by Moriarty.^6^ Considering that alcoholic people may have a high number of treatment interruptions,^2^ our report highlights the importance of a multidisciplinary team approach in the treatment of alcoholism.

The present study also showed that alcoholic patients who received multidisciplinary support were more likely to continue to abstain from alcohol than those who did not. In a previous study, Avila et al. (2008) also found that alcohol‐dependent patients who are referred from internal medicine to psychiatry showed a higher rate of sobriety as well as hospital visit continuity than those who were treated only by psychiatrists.^10^ Furthermore, Mooney and their colleagues^11^ report that a specialized addiction inpatient program consisting of a multidisciplinary team can provide effective continued sobriety. Their report also supported our results that a higher rate of alcohol abstinence can be achieved when healthcare providers of different fields intervene. To identify the high qualified evidence, a larger group of patients with alcohol dependence should be recruited in future studies.

Regarding the outpatient multidisciplinary program, this study showed that alcoholic individuals using only the outpatient alcoholic treatment program could continue to visit the hospital more than those without any multidisciplinary program, but there were no differences in alcohol abstinence among the two groups. Most alcoholic patients are treated during outpatient services such as the intensive outpatient program.^15^, ^16^, ^17^, ^18^ Therefore, the present findings suggest the importance of outpatient services with a multidisciplinary approach for alcoholic patients. Further prospective studies with a large sample size are needed to assess this significance.

Notably, it may be important to understand why a multidisciplinary approach helped the alcohol dependents to continue their hospital visits. Previous studies have shown a significant relationship between substance use disorders and unstable attachment.^12^, ^13^, ^14^ Flores reports that individuals with substance addiction have insecure attachment, and thus attachment theory is useful in the effectiveness of group therapy for substance abuse.^14^ As attachment disorders may contribute to substance dependence, it is often more difficult for alcoholic patients to find someone they can trust, in comparison to other patients. Accordingly, if alcohol‐dependent patients do not trust the medical professionals involved in their treatment, they may soon stop coming to the hospital. People with alcoholism are likely to have insecure attachment to others.^19^ Therefore, it is unlikely that a single supporter's intervention with alcoholic patients can continue without failure. A multidisciplinary approach is a way to expand the circle of support by using supporters who have a good relationship with the patient as a stepping stone. Thus, the more medical professionals are involved, the more likely the patient is to find one professional they can trust. From the perspective of medical professionals, it is a heavy burden for only one specialist to support a patient. If the patient is supported by a multidisciplinary team, they can be treated by professionals with whom they have a good relationship, which can lead to better recovery outcomes.

In this study, there were no differences in abstinence rates between alcoholic patients with and without disulfiram, cyanamide, or acamprosate. A previous randomized controlled study with large samples showed disulfiram's treatment failure to increase the abstinence rate in patients with alcohol dependence. Our results were consistent with the previous study's findings^20^, ^21^, ^22^ even though pharmacotherapy, including disulfiram, acamprosate, and naltrexone, is recommended by the guidelines for treatment of alcoholism.^23^ As various psychosocial interventions for alcoholism such as motivational enhancement therapy, cognitive behavioral therapy, and family therapy have strong positive effects on alcohol abstinence, reduction in problem‐drinking, and alcohol‐related outcomes,^24^ our findings could highlight the effectiveness of a multidisciplinary approach for alcoholic abstinence.

There were no differences in demographic data regarding alcohol abstinence among each alcoholic group with or without the multidisciplinary approach in this study. Previous studies have reported that various demographic factors such as psychiatric comorbidity, family histories of alcoholism, co‐inhabitants drinking alcohol, and the presence of other substance dependence.^25^, ^26^ As the main reason for the difference between our results and the prior findings, the sample size of this study was relatively small. Further studies should be implemented with detailed demographic information to clarify abstinence and alcoholic‐related outcomes.

Regarding differences in characteristics among the two groups, the AUDIT and AST values were significantly lower in the group without the multidisciplinary approach than that with it. This finding may implicate that the alcoholic patients with lower AUDIT or AST values distance themselves from any needs for a multidisciplinary treatment program regarding alcoholism, based on the thought of not hitting rock‐bottom or denying alcoholism. The denial of problem drinking can lead to less motivation to engage in intensive alcohol treatment programs.^27^ The present finding suggests that developmental studies are needed to facilitate alcoholic patients with low AUDIT or mild or normal liver function values, who have problem drinking and require treatment, to participate in multidisciplinary treatment programs.

Regarding the effect of the presence of psychiatric comorbidity on treatment outcome for alcoholism with a multidisciplinary approach, previous studies show that the presence of comorbid psychiatric disorders, including bipolar disorder, is likely to lead to a poor prognosis such as rehospitalization, mortality, and suicide.^28^, ^29^ Our findings suggest that treatment programs for alcoholism with a multidisciplinary approach may be effective for alcoholic patients without neuropsychiatric comorbidities than for patients with those. However, our sample size of alcoholic patients with comorbid psychiatric diseases was relatively small. Hence, further studies with large sample sizes, including comorbid psychiatric patients with alcohol dependence, are needed to reveal the effect of a multidisciplinary treatment program for alcoholism.

There are several limitations to this study. First, the design of this study is retrospective. To obtain more detailed and valid data, a prospective study should be conducted. Second, this study is observational in nature and not a randomized controlled trial (RCT). The differences between the two lie in the level of monitoring and restrictions imposed on the participants' behavior before the evaluation. The degree of standardization of experimental interventions is usually much higher in RCTs. In the future, RCTs will be necessary to examine the effects of multidisciplinary interventions for alcoholism without bias. Finally, this study was conducted in a single hospital and with a small sample. To improve the content of multidisciplinary addiction treatment programs, multicenter studies covering various regions are needed.

In conclusion, this study demonstrates that a multidisciplinary approach for the treatment of alcoholic patients could help reduce the discontinuation of hospital visits among patients with alcohol dependence and improve the level of alcohol abstinence.

## AUTHOR CONTRIBUTIONS

T.K. and T.H. contributed to the conception and design of the study. T.K. led the data collection process. T.H. contributed to the data analysis of this study. H.S., M.I., and S.K. contributed to the execution of the study and were involved in the interpretation of the results. T.K. and T.H. wrote the first draft of the manuscript. All authors reviewed and approved the final manuscript.

## FUNDING INFORMATION

There were no funding source and any specific grants from agencies in the public, commercial, or not‐for‐profit sectors.

## CONFLICT OF INTEREST STATEMENT

Dr. Kurata has received speaker honoraria from or served as a consultant to Otsuka Pharmaceutical Co., Nippon Shinyaku Co., Kyowa Pharmaceutical Industry Co., Sumitomo Dainippon Pharma Co., and Yoshitomiyakuhin Co. Dr. Ishige has received speaker honoraria from Janssen Pharmaceutical K.K. and Sumitomo Dainippon Pharma Co. Dr. Kikuchi has received speaker honoraria from or served as a consultant to Takeda Pharmaceutical Co., Viatris Inc., Sumitomo Dainippon Pharma Co., Meiji Seika Pharma Co. and Shin Nippon Biomedical Laboratories. The other authors declare no conflicts of interest.

## ETHICAL APPROVAL

Approval of the Research Protocol by an Institutional Reviewer Board: This study was approved by the Ethics Committee of Sodegaura Satsukidai Hospital in Sodegaura City, Japan. The present study was conducted in accordance with the Helsinki Declaration.

Informed Consent: The Ethics Committee of Sodegaura Satsukidai Hospital waived the requirement for approval and written informed consent for patient participation, as the present study was retrospective in nature where only anonymized data were used to protect the privacy of the participants by maintaining confidentiality. Information regarding this study was posted in the outpatient department and daycare of Sodegaura Satsukidai Hospital to notify participants of the opt‐out option.

Registry and the Registration No. of the Study/Trial: N/A.

Animal Studies: N/A.

## Data Availability

The database of this study consists of raw data from each participant's medical record at Sodegaura Satsukidai Hospital. Because the data contains potentially identifying participant information, public data sharing will lead to the possibility lead to threatening to participants' privacy. The data that supports the findings of this study is available on request from the corresponding author.
